# Comparative Effectiveness of Transcutaneous Afferent Patterned Stimulation Therapy for Essential Tremor: A Randomized Pragmatic Clinical Trial

**DOI:** 10.5334/tohm.798

**Published:** 2023-10-16

**Authors:** Dingwei Dai, Joaquim Fernandes, Han Kim, Henriette Coetzer

**Affiliations:** 1 CVS Health Clinical Trial Services LLC, Woonsocket, RI, USA; 2 Cala Health, Inc., San Mateo, CA, USA

**Keywords:** transcutaneous afferent patterned stimulation, essential tremor, peripheral neuromodulation, hand tremor, activities of daily living, randomized pragmatic clinical trial

## Abstract

**Background:**

Transcutaneous afferent patterned stimulation (TAPS) is a wrist-worn, non-invasive therapy delivering calibrated stimulation to the median and radial nerves. Previous randomized controlled studies have demonstrated the efficacy and safety of TAPS therapy in some patients with essential tremor (ET), but evidence supporting therapeutic benefits of TAPS versus standard of care (SOC) is lacking. This randomized prospective study evaluated the clinical benefit of adding TAPS treatment to SOC versus SOC alone.

**Methods:**

This randomized pragmatic trial recruited patients from a large health plan’s Commercially Insured and Medicare Advantage population. All 310 patients received a TAPS device and were randomized 1:1 to either one month adding TAPS therapy to usual care (TX arm) or usual care with tremor assessment only (SOC arm). The pre-specified endpoints were changes in tremor power measured by motion sensors on the device (primary) and improvement in Bain & Findley Activities of Daily Living (BF-ADL) upper limb scores (secondary) between TX and SOC in all patients who completed the one-month study.

**Results:**

276 patients completed the one-month study (N = 133 TX, N = 143 SOC). The study met the primary and secondary endpoints, with significantly reduced tremor power in TX compared with SOC (0.017 (0.003) versus 0.08 (0.014) (m/s^2^)^2^; geometric mean (SE); *p* < 0.0001) and greater improvement in the BF-ADL score in TX than SOC (1.6 (0.43) vs 0.2 (0.37) points; mean (SE); *p* < 0.05). No serious device-related adverse events were reported.

**Discussion:**

This trial demonstrates that adding TAPS treatment to SOC significantly improves tremor power and BF-ADLs in patients with ET compared to SOC alone over one month of home use.

**Highlights:**

This study found that adding TAPS treatment to SOC significantly improves tremor power and BF-ADL scores in patients with ET compared to SOC alone over one month of home use. This real-world evidence study suggests that non-invasive TAPS therapy is a safe and valuable treatment option for patients with ET.

## Introduction

Patients living with postural and kinetic hand tremors struggle with key activities of daily living such as eating, drinking, and writing [1,2,3,4]. Most patients with postural and kinetic hand tremors have essential tremor (ET), a condition that affects approximately 7 million Americans and 25 million patients worldwide [5,6]. ET contributes to increased healthcare costs [7,8]. Other less common causes of postural and kinetic hand tremors include Parkinson’s disease, spinocerebellar ataxia, multiple sclerosis, or other conditions [9].

Pharmacological treatments are the primary therapy option for management of ET; however, they are often either ineffective, contraindicated, or associated with side effects that lead to discontinuation for most patients [10,11]. While thalamic deep brain stimulation (DBS) or thalamotomy via radiofrequency or ultrasound relieve tremor for most patients, these devices and procedures carry substantial cost, surgical risk, and adverse effects, including intracranial hemorrhage, paresthesia, and gait disturbances [12,13,14,15,16]. As a result, less than 3% of ET patients proceed with invasive neurosurgical procedures such as DBS [8].

Given the challenge faced by ET patients and the limited effectiveness and high burden/risk of current treatment options, there is an increasing demand for novel, non-invasive, non-pharmaceutical therapy alternatives. Transcutaneous afferent patterned stimulation (TAPS) is a wrist-worn, non-invasive neuromodulation therapy that delivers individually calibrated stimulation to reduce postural and kinetic tremors. TAPS devices were authorized by the U.S. Food and Drug Administration (DEN170028) for the treatment of essential tremor symptoms, recent label expansion includes both ET and postural and kinetic hand tremors in Parkinson’s disease [17,18]. TAPS devices measure each patient’s unique tremor signature and deliver alternating bursts of stimulation pulses to the median and radial nerves at each patient’s tremor frequency [19,20,21,22]. Patients use TAPS to relieve tremor symptoms when needed, typically when patients’ tremors are worse or in preparation for activities requiring hand control.

While previous studies have demonstrated the effectiveness and safety of TAPS in open-label and sham-controlled studies [19,20,21,22,23,24], randomized evidence with home use beyond in-office assessment or in comparison to standard of care remains lacking. The purpose of this study was to evaluate the clinical outcomes of adding TAPS to standard of care over a one-month period of home use.

## Methods

### Study Design

This randomized pragmatic trial recruited patients from the database of a large health insurer (Aetna/CVS Health). All enrolled patients were initially prescribed a TAPS device and then randomized to treatment (TX) or standard of care (SOC) for one month of home use. Patients in the TX arm added TAPS to their physician-recommended care plan while patients in the SOC arm continued with their physician-recommended care plan. After completing one month of SOC, patients in the SOC arm crossed over into TX, and all patients continued with treatment for an additional 11 months of an ongoing open-label 12-month study phase (Figure S1).

The study was approved by the Sterling institutional review board (Atlanta, GA, USA) prior to implementation of study procedures, and registered with ClinicalTrials.gov (NCT05540626). Electronic informed consent was obtained from all study participants. This report follows the Consolidated Standards of Reporting Trials (CONSORT) reporting guideline for randomized clinical trials [25].

### Patient Selection

**Figure 1:**
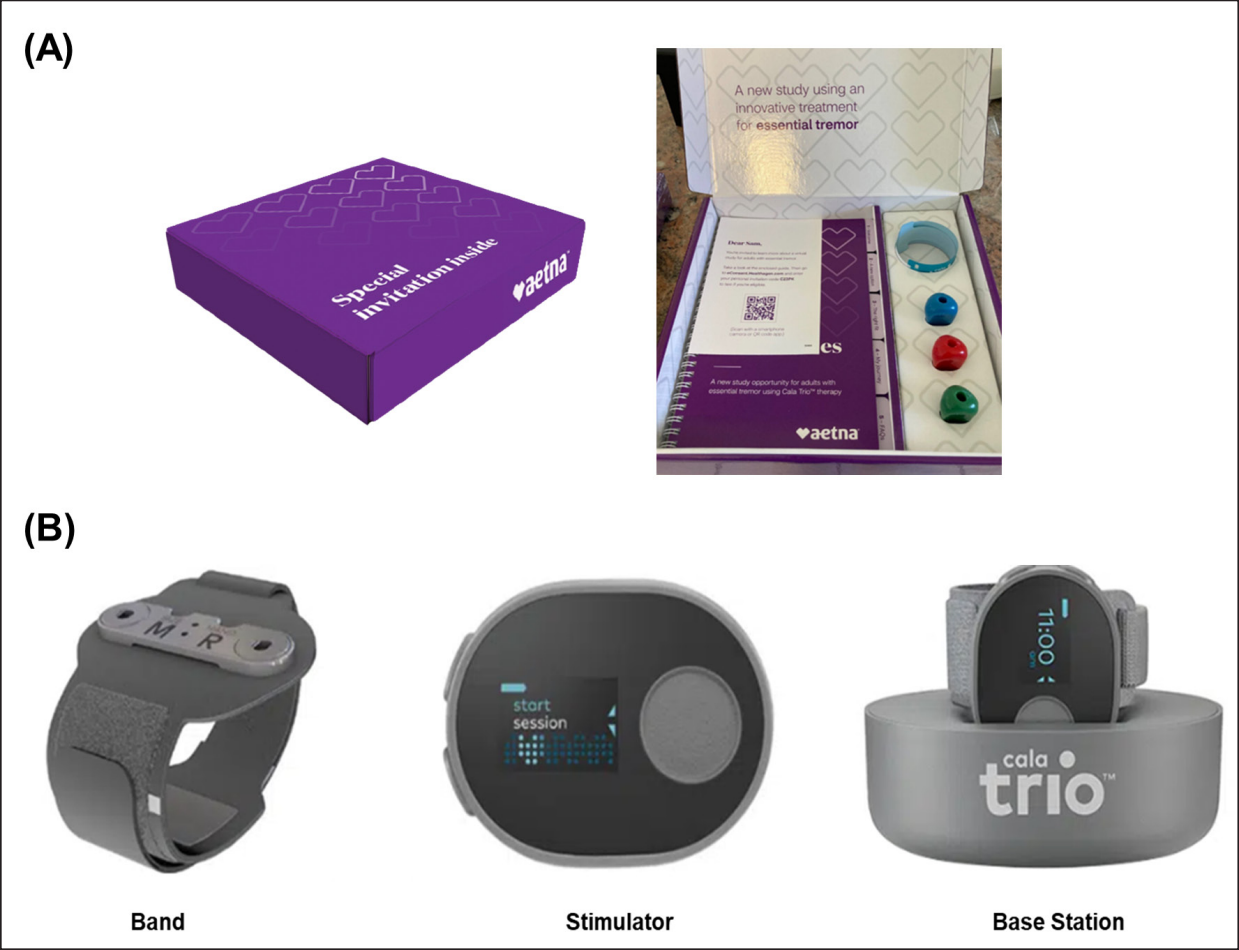
**Recruitment Kit and TAPS Device Components. (A)** Custom Recruitment Box tailored to ET patients included: Journal, Cala Trio supplied wrist measurement tool, Study Invitation including QR code directing to custom eConsent site, custom personal invitation code. **(B)** The Cala Trio™ device is comprised of a band, stimulator, and base station. The band is a wristband with embedded electrodes for delivering TAPS to the median and radial nerves. The stimulator snaps into the band to deliver an individualized stimulation pattern to the median and radial nerves. The base station charges the device and uploads device data to a secure cloud platform.

Candidate patients were identified in the claims databases of a major payor by screening patients claims with the study’s inclusion and exclusion criteria. Representatives of the health plan reached out to patients by email or direct mail and invited patients to participate in the study. Patients were sent a custom recruitment box tailored to ET (Figure 1A) and directed to a study website built and managed by CVS Clinical Trial Services for additional study information and screening. Patients that passed the screening and were interested in participating in the study were asked to sign an informed electronic consent form and a Health Insurance Portability and Accountability Act (HIPAA) waiver allowing study personnel to contact their physician via phone or fax to request a prescription for TAPS therapy. The patient’s physician confirmed an ET diagnosis when they signed the prescription form. Patients who failed the screening were excluded from the study.

The inclusion criteria for the study were: (1) patients diagnosed with ET defined as having at least two medical claims with an ET diagnosis code (International Classification of Diseases, Tenth Revision, Clinical Modification (ICD-10-CM): G25.0) at least seven days apart in the last three years OR one claim with an ET diagnosis code followed by at least one dispensed pharmaceutical treatment that can be used for ET; (2) patients aged 22 years older; (3) patients with fully insured commercial health plan or/and Medicare Advantage with medical and pharmacy health insurance benefits for at least 12 months before the enrollment; and (4) patients able to sign informed consent.

The exclusion criteria for the study were: (1) patients with a diagnosis of Parkinson’s disease (ICD-10-CM: G20.x), Alzheimer’s disease/dementia (ICD-10-CM: F00.x–F03.x, F05.x, G13.8, G30.x, G31.1, G31.83, G94.x, R41.81), epilepsy (ICD-10-CM: G40.x), thyroid disorders (ICD-10-CM: E00.x–E03.x, E06.5, E07.9, E89.0, or thyroid hormone prescription); (2) patients with an implanted electronic medical devices such as a pacemaker, defibrillator, or deep brain stimulator; (3) patients who had undergone an ET-related neurosurgery including thalamotomy, gamma-knife radio surgical thalamotomy, or magnetic resonance-guided focused ultrasound; (4) patients who had used botulinum toxin as a therapeutic injection in the upper limb during the last six months; (5) patients who were pregnant during the enrollment period or planned to become pregnant during the course of the study; or (6) patients with any hand skin lesions at the stimulation site.

### Patient Randomization

Participants receiving a physician prescription were randomized 1:1 to the TX or SOC arm following a randomization list generated by a study statistician. Randomization was stratified by insurance type (commercial health insurance or Medicare Advantage plan) to ensure a balanced distribution of ages between the TX and SOC arms.

### Patient Enrollment and Training

Patients in both arms scheduled an appointment with patient support personnel at Cala Health to complete enrollment and training on proper use of the device (Cala Trio™, Cala Health, San Mateo, CA, USA). All patients received a standard TAPS delivery containing three components sent directly to their home: (1) a wrist-worn stimulator that delivers an individualized stimulation pattern to the median and radial nerves, and measures patients’ tremor during postural holds with an on-board accelerometer, (2) a detachable wristband with multiple embedded electrodes configured to target the median and radial nerves, and (3) a cloud-connected base station which charges the stimulator and securely transmits all device data to a cloud platform (Figure 1B).

All patients in the TX arm completed standard TAPS training with the Cala Trio device. The training included instructions to perform postural holds before and after stimulation sessions for measuring tremor power, self-administer 40-minute stimulation sessions as needed to control their tremor, report complaints, and a reminder to continue with any other ET-related care and comorbidity management recommended by their physician. Patients in the TX arm were instructed to use the device as-needed according to the instructions for use, which states that “Cala Trio therapy should be applied when temporary relief of hand tremor is desired (i.e., before activities involving your hands such as meals or writing).” Patients in the SOC arm were given instructions to measure tremor power once a day with the Cala Trio device, without delivering stimulation, for one month. Additional study-specific training was given to both arms and included instruction on completing a Bain & Findley Activities of Daily Living (BF-ADL) upper limb rating assessment scale at baseline and one month, and notifying study personnel of any therapy modifications, as applicable. The wording used on the BF-ADL survey for the upper limb tasks and ratings matched that from the original publication [28]. Additional details on the design of the study after the first month can be found in the supplementary materials.

### Data Collection

Demographic and clinical data were collected either directly from patients or abstracted from the patients’ medical and pharmacy claims in the health plan’s administrative claims database and entered into the electronic Case Report Form (eCRF) by trained study personnel. Comorbid conditions in the medical claims were identified using previously described methods based on ICD-10-CM [7,26]. Charlson Comorbidity Index (CCI) and age adjusted CCI (ACCI), two validated indexes that summarize disease burden and predict mortality and high health care costs were also calculated [7,27]. Baseline ET-related medications and psychiatric medications prescribed in the 12 months prior to study enrollment were identified from pharmacy claims in the payor’s claims database based on national drug codes [7]. For the TX arm, TAPS device usage and motion data were retrieved from device logs, which contained timestamps of all sessions and the accelerometer measurements during postural holds prompted by the device immediately before and after stimulation sessions. Similarly, for the SOC arm, motion data was retrieved from device logs for the single, daily postural hold measurement. Tremor power was calculated from accelerometry measurements by integrating power spectral density around the peak tremor frequency in the 4–12 Hz band, and tremor power improvement ratio was calculated as the ratio of pre-stimulation to post-stimulation tremor power for each stimulation session. These calculations followed methods established in previous publications, as did quality assessments used to remove invalid therapy sessions [20,21,22].

The BF-ADL upper limb scores were collected using patient surveys administered on the e-consent platform at baseline and the end of one month. The eight BF-ADL upper limb tasks assessed were to (1) use a spoon to drink soup, (2) hold a cup of tea, (3) pour milk from a bottle, (4) dial a telephone, (5) pick up change, (6) insert an electric plug, (7) unlock front door, and (8) write a letter. Each task was rated on a scale of 1 (“able to do the activity without difficulty”), 2 (“able to do the activity with little effort”), 3 (“able to do the activity with a lot of effort”), to 4 (“cannot do the activity by yourself”) [20,28]. The scores were aggregated across the eight tasks at baseline and at the end of one month. BF-ADL tasks not involving the upper limb were not included in the survey.

Adverse events (AEs) were self-reported by patients and were entered into the electronic Case Report Form (eCRF) by trained study personnel.

### Statistical Analyses

Analyses were performed according to a prespecified statistical analysis plan. Descriptive analyses present distributions as frequency (%) for categorical variables and means with standard deviations (SDs) or medians with interquartile ranges (IQR), as appropriate, for continuous variables. Differences between the study arms and between the subgroups (age, gender, severity) were tested using either a two-sample t-test or the Wilcoxon rank-sum test for continuous variables and Pearson x^2^ test for categorical variables. All statistical analyses were conducted using SAS version 9.4 statistical software (SAS Institute Inc., Cary, NC). All *p* values are two-sided, with *p* < 0.05 considered statistically significant. AEs were summarized using frequency counts and percentages. Holm-Bonferroni corrections were applied as needed for multiple comparisons.

The pre-specified modified intention-to-treat (mITT) analysis population for the primary and secondary endpoints included all the patients who completed one month of study. The per-protocol (PP) analysis population included all participants from the mITT population who adhered adequately to the protocol. In the SOC arm, participants who purposefully or accidentally initiated more than one therapy session greater than or equal to 5 minutes in duration during the month were excluded from PP analysis. In the TX arm, participants who completed only one TAPS session or no TAPS sessions during the month were excluded from PP analysis. The rationale is that these TX patients would not have complied with the study protocol of using TAPS as needed beyond the training session. If a TX patient completed at least two TAPS sessions of at least 5 minutes duration during the month, they were included in the PP analysis.

### Primary Endpoint

The study’s pre-specified primary endpoint was the difference in median tremor power for patients in the TX versus SOC arm in the mITT, with statistical significance evaluated using a Wilcoxon rank-sum test. Tremor power for each patient in the TX arm was the median tremor power measured during postural holds performed after each self-administered TAPS session over the month of home use. Tremor power for each patient in the SOC arm was the median tremor power measured during postural holds performed daily over the month of home use. Tremor power for both arms was summarized as a geometric mean and geometric standard error, equivalent in range to the mean and standard error of log-transformed data, because tremor power is logarithmically distributed.

### Secondary Endpoint

The study’s pre-specified secondary endpoint was the difference in BF-ADL upper limb score improvement from baseline to the end of the first month for patients in the TX arm versus the SOC arm in the mITT, with statistical significance evaluated using a two-sample t-test. BF-ADL upper limb scores for both arms were summarized using the mean and standard deviation.

### Exploratory Endpoints

Pre-specified exploratory analyses of BF-ADL scores in the mITT included assessment of improvement in BF-ADL score from baseline to one month within each arm, and improvement in individual BF-ADL tasks from baseline to one month within each arm. A two-sample t-test with Holm-Bonferroni corrections for multiple hypothesis testing was performed to compare the total and per-task changes in BF-ADL scores from baseline to the end of the month between the TX arm and SOC arm.

Pre-specified exploratory analysis of tremor power in the mITT included assessment of improvement within the TX arm. Each patient’s improvement was defined as the median tremor power improvement ratio (TPIR) across all of that patient’s TAPS sessions, where TPIR was the ratio of tremor power before stimulation to after stimulation [20]. An improvement ratio of 1 signified no change in tremor power, while a ratio greater than 1 indicated tremor improvement (i.e., a reduction in tremor power), such as a 2-fold improvement ratio corresponding to a 50% tremor power reduction and a ratio less than 1 indicated tremor worsening (i.e., an increase in tremor power).

### Severe ET Subgroup Analyses

A subgroup of more severe patients was defined as patients who scored a 3 or 4 on at least one of four BF-ADL tasks associated with eating, drinking, or writing at baseline. These included: (1) write a letter, (2) use a spoon to drink soup, (3) hold a cup of tea, and (4) pour milk from a bottle. The primary and secondary endpoints were analyzed within this subgroup and further stratified by age.

### TAPS Device Usage Analysis

Usage in the TX group, as pre-specified, was assessed as the number of therapy sessions per week, total days per week with at least one therapy session, and the average number of therapy sessions per day on days therapy was used. Usage assessments were further stratified by age and gender.

### Sample Size Calculation

The study sample size was calculated to sufficiently power the study’s primary endpoint. A 25% patient attrition was assumed based on the one-month dropout rate in a previous study [20]. It was determined that to detect 50% tremor power reduction through TAPS therapy with a significance level (α) of 5% and a power (1 – β) of 90%, the trial needed to recruit 300 patients with 1:1 ratio, 150 per randomization arm (TX or SOC).

## Results

### Study Population and Baseline Characteristics

**Figure 2:**
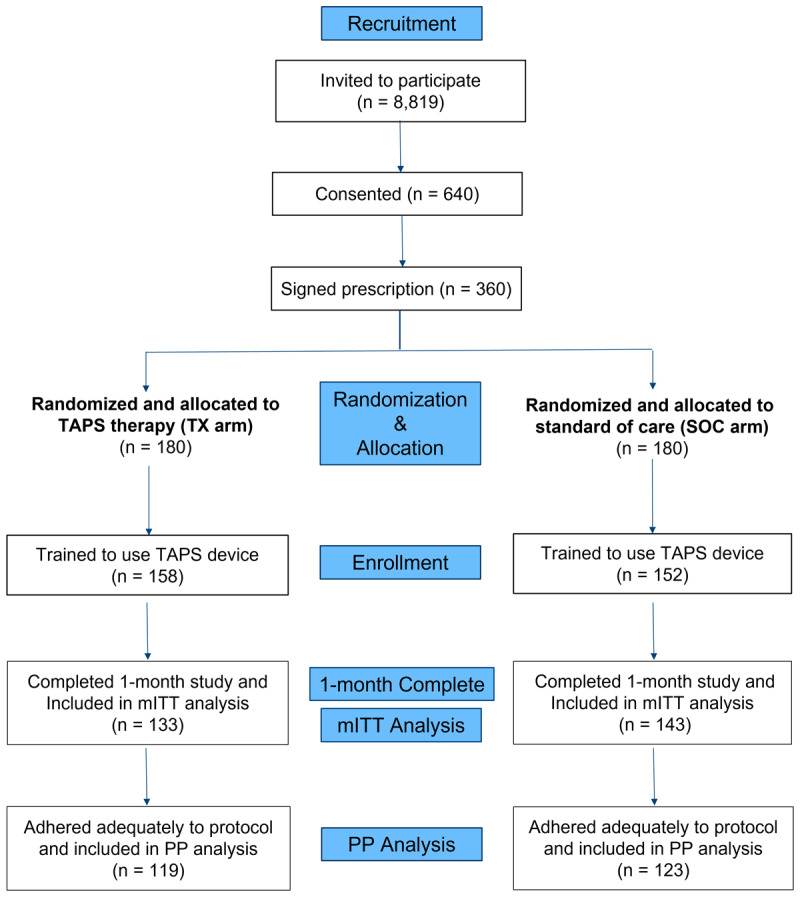
**Enrollment flow chart.** Of the 310 participants enrolled, 276 completed the one-month study and were included in the modified intention-to-treat (mITT) analysis. Within the mITT population, 242 participants were adequately adherent to protocol and were included in the per-protocol (PP) analysis.

310 patients were enrolled in the study between May 2021 and February 2023 and randomized to treatment (N = 158) or standard of care (N = 152) (Figure 2). Enrolling the study required inviting 8,819 patients to participate, of which 901 (10.2%) patients took the self-screener, and 753 (8.5%) passed this screening. Reasons for screening failure included 9 patients with a diagnosis of Parkinson’s disease, 15 patients with implanted electronic medical devices, 2 with skin lesions at the stimulation site, 5 with botulinum toxin injections in the last 6 months, 5 with seizures, 38 with thyroid problems, and 74 with memory difficulty. 640 patients (7.3%) consented, of which 360 (4.1%) received a signed prescription for TAPS therapy from their physicians and were randomized to TX or SOC, and 310 (3.5%) were enrolled. Of the 50 patients who received a signed prescription but were not enrolled in the study, 3 were diagnosed with Parkinson’s disease, 6 had health problems or surgeries, 3 were no longer Aetna members, 3 were no longer interested in the study, and 35 were not responsive to emails or calls.

276 (133 TX, 143 SOC) patients completed one month of the study between May 2021 and March 2023 and were included in the mITT population. Reasons for discontinuation included insufficient tremor relief (N = 10), difficulty in using the device or fitting it into their lifestyle (6), health condition interfering with participation (3), contraindication (2), PD diagnosis (1), changed insurance (1), lost device (1), tremor too minor at baseline (1), and unknown/patient did not respond (9).

242 patients (119 TX; 123 SOC) in the mITT were compliant with the protocol and thus included in the PP analysis. 20 SOC patients with more than one valid TAPS stimulation (≥5 minutes in duration) and 14 TX patients with only one valid TAPS stimulation were excluded from the PP population.

There were no statistically significant differences in sociodemographic and baseline clinical characteristics between patients in the TX and SOC arms (Table 1). Patients were 66% male, 70% had received ET-related pharmacotherapy within 12 months prior to the enrollment (including 27% with primidone and 21% with propranolol) and had 7 (5 – 9) comorbidities (median (interquartile range)). Patients’ mean (SD) age was 68.2 (11.1) years, and 74% were age 65 or older. 169 (82%) of the patients 65 or older and 55 (77%) of the patients under 65 met the criteria for the severe subgroup analysis that required having at least one eating, drinking, or writing task be rated a 3 or 4 on the BF-ADL score.

**Table 1:** Baseline sociodemographic and clinical characteristics of the mITT population.


CHARACTERISTICS	TOTAL	TX	SOC	*p* VALUE

	(N = 276)	(N = 133)	(N = 143)	TX VS SOC

***Sociodemographic characteristics***				

**Age (years)**, Mean (SD)	68.21 (11.09)	67.77 (11.71)	68.61 (10.51)	0.21

**Age Group (years)**, N (%)				0.62

22–44	11 (3.99)	7 (5.26)	4 (2.80)	

45–64	60 (21.74)	31 (23.31)	29 (20.28)	

65–74	124 (44.93)	56 (42.11)	68 (47.55)	

≥75	81 (29.35)	39 (29.32)	42 (29.37)	

**Gender**, N (%)				0.31

Male	183 (66.30)	84 (63.16)	99 (69.23)	

Female	93 (33.70)	49 (36.84)	44 (30.77)	

**Race**, N (%)				0.31

Black	5 (1.81)	1 (0.75)	4 (2.80)	

White	233 (84.42)	115 (86.47)	118 (82.52)	

Hispanic	3 (1.09)	1 (0.75)	2 (1.40)	

Asian	3 (1.09)	3 (2.26)	0	

More than one race	3 (1.09)	2 (1.50)	1 (0.70)	

Other	4 (1.45)	1 (0.75)	3 (2.10)	

Unknown	25 (9.06)	10 (7.52)	15 (10.49)	

**Payers**, N (%)				0.37

Commercial insurance	88 (31.88)	46 (34.59)	42 (29.37)	

Medicare Advantage	188 (68.12)	87 (65.41)	101 (70.63)	

***Clinical characteristics***				

**Comorbidities**				

**Charlson Comorbidity Index (CCI)**, Mean (SD)	2.23 (2.50)	2.06 (2.54)	2.38 (2.46)	0.73

**Age-adjusted CCI**, Mean (SD)	4.78 (2.94)	4.58 (3.00)	4.96 (2.87)	0.62

**Number of comorbidities**				0.99

Mean (SD)	7.20 (3.48)	7.26 (3.56)	7.15 (3.42)	

Median (IQR)	7 (5–9)	7 (4–9)	7 (5–9)	

**Psychiatric conditions**, N (%)				

Anxiety	87 (31.52)	40 (30.08)	47 (32.87)	0.62

Depression	69 (25.00)	34 (25.56)	35 (24.48)	0.83

Substance use disorders	28 (10.14)	11 (8.27)	17 (11.89)	0.32

Stress and adjustment disorders	20 (7.25)	9 (6.77)	11 (7.69)	0.77

**Medication use**, N (%)				

ET medications				

Primidone	74 (26.81)	41 (30.83)	33 (23.08)	0.15

Propranolol	58 (21.01)	22 (16.54)	36 (25.17)	0.10

Topiramate	23 (8.33)	10 (7.52)	13 (9.09)	0.64

Gabapentin	41 (14.86)	20 (15.04)	21 (14.69)	0.93

Other beta blockers	143 (51.81)	73 (54.89)	70 (48.93)	0.32

Other benzodiazepines	15 (5.43)	10 (7.52)	5 (3.50)	0.19

Alprazolam	21 (7.61)	12 (9.02)	9 (6.29)	0.50

Clonazepam	12 (4.35)	5 (3.76)	7 (4.90)	0.77

Any ET related medication	193 (69.93)	96 (72.18)	97 (67.83)	0.43

Number of ET-related Medications				0.28

None	83 (30.07)	37 (27.82)	46 (32.17)	

1	61 (22.10)	26 (19.55)	35 (24.48)	

2	83 (30.07)	47 (35.34)	36 (25.17)	

3	39 (14.13)	20 (15.04)	19 (13.29)	

≥4	10 (3.62)	3 (2.26)	7 (4.90)	

***Tremor Characteristics***				

Baseline BF-ADL severity*, N (%)				

BF-ADL severity ≥2	262 (94.93)	128 (96.24)	134 (93.71)	0.41

BF-ADL severity ≥3	224 (81.20)	112 (84.21)	112 (78.32)	0.22

BF-ADL severity = 4	75 (27.17)	34 (25.56)	41 (28.67)	0.59


* BF-ADL tasks associated with eating, drinking, or writing (i.e., use a spoon to drink soup; hold a cup of tea; pour milk from a bottle; write a letter). Patients were classified as having at least one of these 4 BF-ADL tasks with a score of ≥2, ≥3, or = 4. ** See Supplementary Materials (Table S1) for additional sociodemographic and clinical characteristics.

### Primary Endpoint

**Figure 3:**
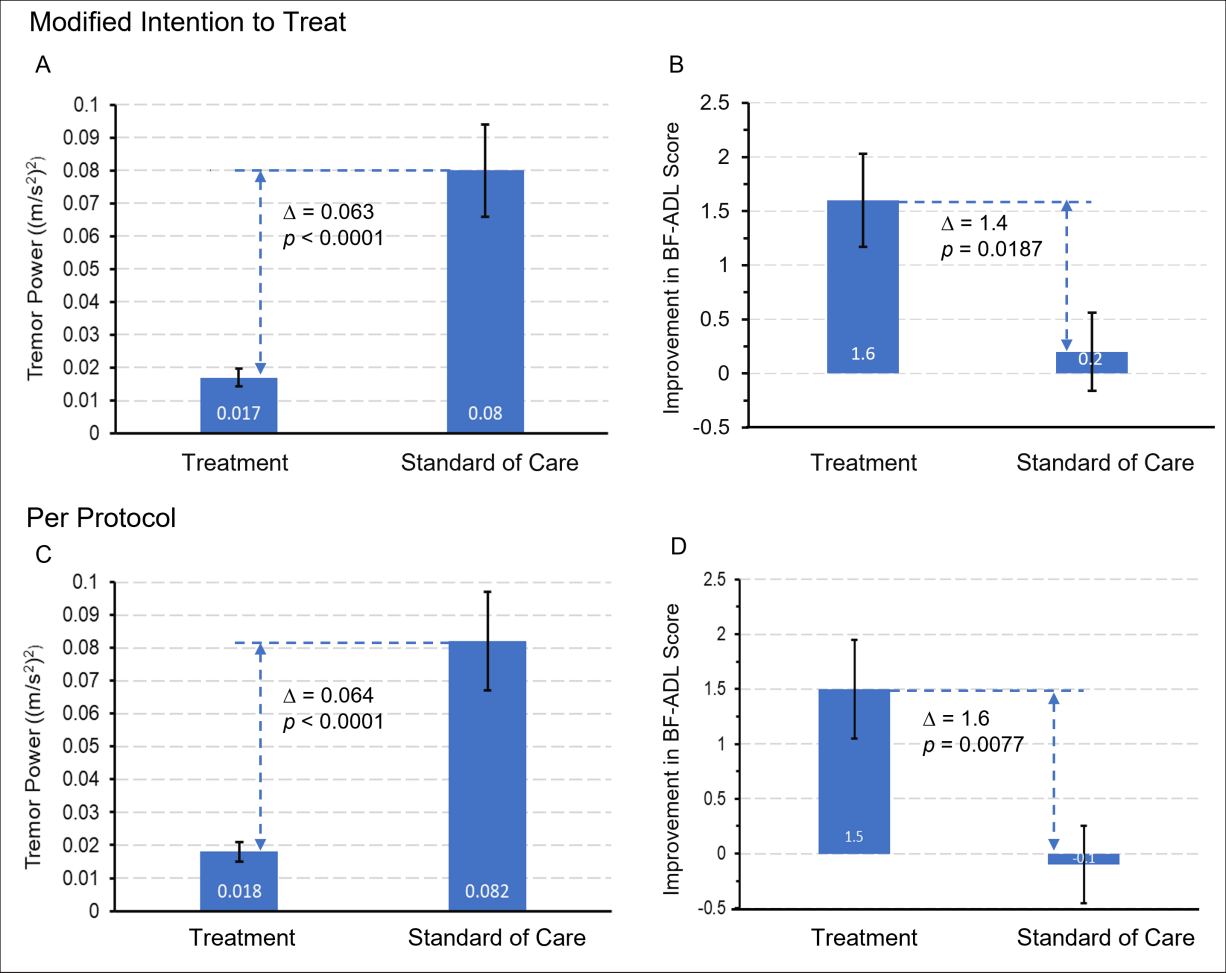
**The study met its primary and secondary endpoints.** (**A** and **C**) Patients in the TX arm had significantly lower (improved) tremor power than patients in the SOC arm in the mITT (primary endpoint, *p* < 0.0001) and PP (*p* < 0.0001) populations. 133 TX and 143 SOC patients were included in the mITT analysis while 119 TX and 123 SOC were included in the PP analysis. (**B** and **D**) Patients in the TX arm had significantly greater improvement in BF-ADL scores than SOC in the mITT (secondary endpoint, *p* = 0.0187) and PP populations (*p* = 0.0077, paired). 134 of the 276 patients and 114 of the 242 patients completed the BF-ADL ratings at baseline and one month for the mITT and PP populations respectively.

The primary endpoint was met in both the mITT and PP populations. Tremor power in the TX arm (0.017 ± 0.003 (m/s^2^)^2^) was significantly lower than tremor power in the SOC arm (0.08 ± 0.014 (m/s^2^)^2^) in the mITT (*p* < 0.0001) (Figure 3A). The primary endpoint was also met in the PP population, with tremor power in the TX arm (0.018 ± 0.003 (m/s^2^)^2^) also significantly lower than tremor power in the SOC arm (0.082 ± 0.015 (m/s^2^)^2^) (*p* < 0.0001) (Figure 3C).

### Secondary Endpoint

The secondary endpoint was also met in both the mITT and PP populations. 134 of the 276 patients completed the BF-ADL ratings at baseline and one month. The changes in BF-ADL score from baseline to one month in the TX arm (1.6 ± 0.43, N = 51) were significantly greater than the changes observed in the SOC arm (0.22 ± 0.37, N = 83) (*p* = 0.0187) (Figure 3B). The secondary endpoint was also met in the PP population, 114 of the 242 patients in the PP population completed the BF-ADL ratings at baseline and one month. The changes in BF-ADL score from baseline to one month in the TX arm (1.5 ± 0.45, N = 47) were significantly greater than changes observed in the SOC arm (–0.1 ± 0.37, N = 67) (*p* = 0.0077) (Figure 3D).

### Exploratory Endpoints

**Figure 4:**
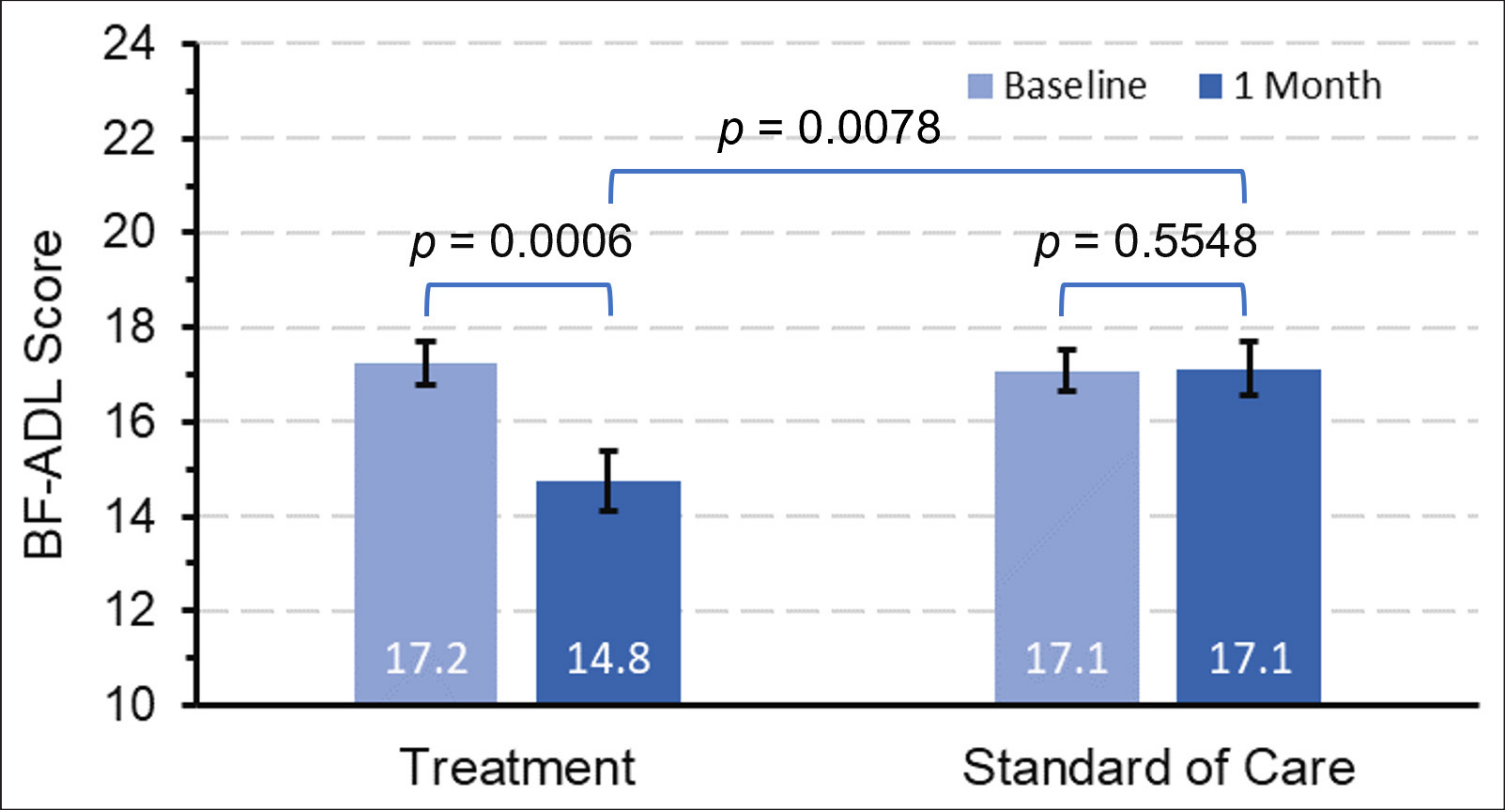
**BF-ADL score at baseline and the end of one month.** BF-ADL scores at one month were lower (i.e., improved) in the TX arm than the SOC arm (*p* = 0.0078, unpaired).

On average, the BF-ADL score improved significantly from baseline to one month by 2.4 points in the TX arm (*p* = 0.0006) whereas no significant improvements were observed in the SOC arm (*p* = 0.55) (Figure 4). This analysis differs from Figure 3B in that BF-ADL scores at baseline and one month are no longer required to be paired; therefore, Figure 4 is more reflective of the overall population BF-ADL scores. Baseline BF-ADL scores were available for N = 124 in TX and N = 130 in SOC; and one month BF-ADL scores were available for N = 55 in TX and N = 87 in SOC. The improvements reported in Figure 4 are the difference between the unpaired population average BF-ADL scores at one month between the TX and SOC arm.

**Figure 5:**
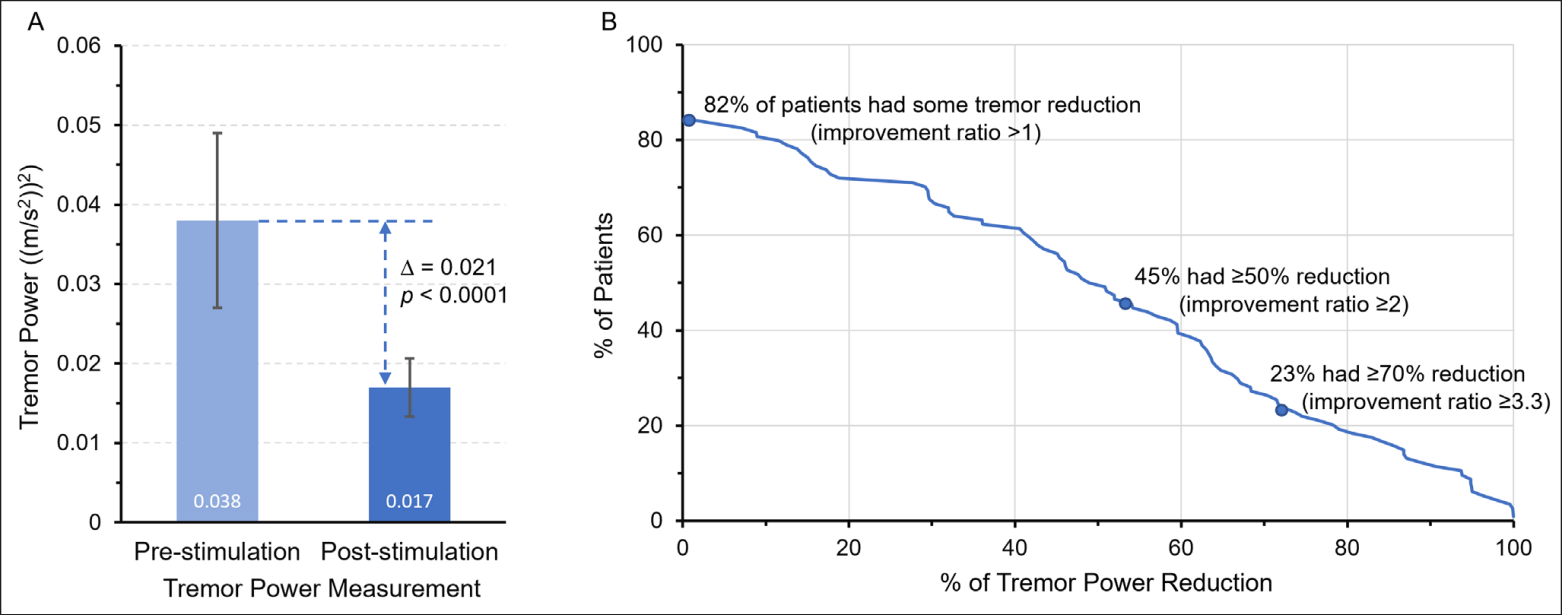
**Additional analyses of tremor power in the TX arm. (A)** Tremor power in the TX arm was significantly lower (improved) at post-stimulation compared to pre-stimulation (*p* < 0.0001; geometric mean ± geometric standard error). **(B)** Percentage of patients with different levels of median tremor power reduction over all sessions performed. 109 of 133 patients (82%) in the TX arm had some tremor power improvement, 60 of 133 patients (45%) had greater than 50% tremor power improvement, and 30 of 133 (23%) had greater than 70% tremor power improvement.

The average median tremor power in those using TAPS therapy (TX) improved from 0.038 ± 0.011 (m/s^2^)^2^ pre-stimulation to 0.017 ± 0.004 (m/s^2^)^2^ post-stimulation (*p* < 0.0001) (Figure 5A). TAPS therapy used over one month resulted in 45% of patients experiencing a ≥50% tremor power reduction (≥2 improvement ratio in tremor power, indicating at least 2-fold improvement from pre- to post-tremor power), and 25% experiencing a ≥70% reduction (≥3.3 improvement ratio in tremor power) (Figure 5B).

There were no significant differences in pre-stimulation tremor power between the TX and SOC arms at initial-measurement baseline (TX: 0.046 ± 0.008 (m/s^2^)^2^, SOC: 0.048 ± 0.009 (m/s^2^)^2^, *p* = 0.7585). Yet, over the first month, the pre-stimulation tremor power in the TX arm (0.038 ± 0.011 (m/s^2^)^2^) was significantly lower than the tremor power in the SOC arm (0.08 ± 0.014 (m/s^2^)^2^), *p* = 0.0018).

### Severe ET Subgroup Analysis

TAPS significantly improved tremor power in the severe subgroup, in which tremor power was 0.02 (0.003) (m/s^2^)^2^ in the TX arm and 0.097 (0.019) (m/s^2^)^2^ in the SOC arm (*p* < 0.0001) (Table 2). Analysis of the median tremor improvement per-patient demonstrated that 83% of patients experienced some tremor power reduction, while 46% of patients experienced at least 50% reduction. The change in BF-ADL score from baseline to one month was also larger in the TX arm (2.21 (0.49)) than the SOC arm (0.35 (0.43)) (mean (SE) improvement; *p* = 0.0079 (Table 2).

**Table 2:** Severe subgroup analyses of primary and secondary endpoints between TX and SOC arm in the mITT population*


	AGE (YEARS)		

	AGE < 65	AGE ≥ 65	ALL AGES

Tremor power geometric mean, N, *p*-value	TX = 0.022 (N = 30) SOC = 0.082 (N = 25) *p* = 0.0047	TX = 0.021 (N = 82) SOC = 0.10 (N = 87) *p* < 0.0001	TX = 0.022 (N = 112) SOC = 0.097 (N = 112) *p* < 0.0001

Improvement in BF-ADL score, N, *p*-value**	TX Δ = 2.18 (N = 11) SOC Δ = 1.21 (N = 19) *p* = 0.42	TX Δ = 2.21 (N = 28) SOC Δ = 0.025 (N = 47) *p* = 0.0096	TX Δ = 2.21 (N = 39) SOC Δ = 0.35 (N = 66) *p* = 0.0079


* Severe patients were classified as having at least one of 4 tasks which impacts eating, drinking, or writing BF-ADL task ≥3. ** Includes patients with complete BF-ADL scores from baseline and at one month.

Moreover, patients in the severe subgroup that were aged 65 and above exhibited a 5-fold greater improvement in tremor power in the TX arm than the SOC arm (*p* < 0.0001) and a 2.2-point greater change in BF-ADL score improvement in the TX arm than the SOC arm (*p* = 0.0096). Alternatively, patients below the age of 65 experienced a 4-fold greater improvement in tremor power in the TX arm than the SOC arm (*p* = 0.0047) and did not experience a significantly greater change in BF-ADL scores (*p* = 0.42) (Table 2).

### TAPS Device Usage and Subgroup Analysis by Age Group and Gender

Patients 65 years and older used the TAPS device more frequently than younger patients (4.9 vs 3.0 sessions per week; *p* = 0.0194), but these two age subgroups did not have significantly different tremor power improvement (3.2 vs 4.4 median TPIR, *p* = 0.2841) (Table 3). Female and male patients used the TAPS device similarly (4.6 vs 4.2 sessions per week; *p* = 0.5740) and did not have significantly different tremor power improvement (3.0 vs. 3.8 median TPIR, *p* = 0.4541).

**Table 3:** TAPS device usage and effectiveness by age group and gender in the TX arm.


NUMBER OF SUBJECTS	ALL PATIENTS	AGE (YEARS)			GENDER		

		<65	≥65	*p* VALUE	FEMALE	MALE	*p* VALUE
	
	N = 1 33	N = 38	N = 95		N = 49	N = 84	

**Usage patterns**, Mean (SD)							

Number of sessions per week	4.34 (4.12)	3.03 (2.94)	4.87 (4.41)	0.0194	4.61 (5.12)	4.19 (3.43)	0.5740

Number of days per week with at least one session	3.00 (2.17)	2.28 (1.84)	3.29 (2.23)	0.0142	2.95 (2.26)	3.03 (2.14)	0.8416

Number of sessions per day on days when therapy used	1.32 (0.49)	1.25 (0.41)	1.35 (0.52)	0.3198	1.38 (0.68)	1.29 (0.35)	0.3446

**Tremor power improvement ratio**^1^, Mean (95% CI)							

Improvement ratio, all sessions	3.51 (2.46–4.56)	4.43 (2.48–6.36)	3.16 (2.20–4.10)	0.2841	2.98 (1.77–4.20)	3.81 (2.30–5.32)	0.4541

**Improvement**^2^ **in BF-ADL score**, Mean (SD)							

Improvement in BF-ADL score	1.59 (3.07)	1.81 (1.91)	1.48 (3.50)	0.0147	1.57 (2.27	1.59 (3.35)	0.1355

**BF-ADL individual task improvements**^2^ **in patients with ≥ 3 at baseline for each task**, Mean (SE)							

Pour milk from a bottle	0.92 (0.15) *(N = 24, p < 0.0001)*						

Insert an electric plug	0.80 (0.21) *(N = 20, p = 0.0013)*						

Hold a cup of tea	0.73 (0.11) *(N = 40, p < 0.0001)*						

Dial a telephone	0.70 (0.18) *(N = 20, p = 0.0009)*						

Use a spoon to drink soup	0.61 (0.11) *(N = 46, p < 0.0001)*						

Unlock front door	0.50 (0.20) *(N = 16, p = 0.0271)*						

Pick up change	0.37 (0.19) *(N = 19, p = 0.0691)*						

Write a letter	0.31 (0.08) *(N = 62, p = 0.0006)*						


^1^ Device-measured outcome, improvement ratio was defined pre-stimulation tremor power divided post-stimulation tremor power. ^2^ BF-ADL improvement, BF-ADL score changes defined as BF-ADL score at the end of one month minus BF-ADL score at baseline multiplied by –1. A positive value indicates improved ADL from baseline over one month.

### Adverse Events

Four patients (3%) reported temporary wrist skin irritation, sores, discomfort, or dizziness including unpleasant stimulation during the one-month study period. All these events were resolved without professional medical attention. No TAPS therapy-related serious adverse events reported.

## Discussion

This was the first randomized pragmatic clinical trial comparing TAPS to SOC in patients with ET, as well as the first randomized TAPS study that included use of TAPS at-home. The study demonstrated that adding TAPS therapy to SOC significantly improved tremor power and BF-ADLs compared to SOC alone during one month of home use, in both the mITT and PP populations (Figure 3). These results expand the previous randomized studies indicating that TAPS provided meaningful and significant tremor relief beyond a single session [19,23]. ET patients consistently rank ADL improvement as their most important area of therapeutic needs [22]. This study demonstrated that the TX group experienced significantly greater improvements in BF-ADL than the SOC group. Furthermore, 6 of 8 individual tasks in BF-ADL significantly improved in the TX group, while none of these tasks improved in the SOC group (Table S2). Subgroup analysis of the proportion of patients rated “Severe” or “Moderate” improved from 54% at baseline to 27% at the end of one-month in the TX group, whereas almost no improvement was observed in the SOC group during the one-month study period (Figure S3). These findings are consistent with previous randomized sham-controlled trial [19] and an at-home prospective study [20].

This study demonstrated how motion sensors can be used to measure tremor power over extended home-use and as an endpoint to evaluate the effectiveness of TAPS therapy or other interventions for ET in clinical trials [20,21,29,30]. ET severity is typically measured in clinic settings using subjective clinician rating scales that are not well-suited for measuring severity throughout the day.

Motion sensors have been widely used to quantify tremor severity in ET patients [29,30,31,32,33] and previous studies using the same wrist-worn accelerometer as the current study demonstrate a significant correlation between clinical tremor rating and average tremor power from accelerometry for postural tremor [20,21]. Despite the consensus that motion sensors are well suited to capture tremor, it may be difficult to compare the absolute tremor power values across studies due to methodological differences. For example, the tremor accelerations measured from the middle finger for ET patients ranged from 17 to 196 cm/s^2^ with a mean of 63.4 cm/s^2^ in a previous study [31]. In contrast, the tremor power reported in the current study, 0.08 (m/s^2^)^2^ (acceleration: 28 cm/s^2^) is the geometric mean of the SOC group, which would be lower but a more accurate measure for metrics following log-normal distribution in comparison to arithmetic mean. Second, ET patients taking any tremor medications that might affect tremor were excluded in a previous study [31], whereas this study included patients who were taking medications. Thus, the lower tremor power and acceleration observed in the current study is likely due in part to the effect of tremor-related medication. Regardless, differences may exist between how patients perceive tremors during their daily routines and the quantified tremor amplitude and severity ratings from motion sensors and clinician-rated scales [33].

Tremor power improvement measured using motion sensors in this study was slightly lower than previously reported clinical trial findings. In this study, 45% of patients demonstrated tremor power improvement greater than 2-fold (Figure 5B). Results were similar in the severe ET subgroup, in which 83% of patients demonstrated tremor power improvement, and 46% demonstrated tremor power improvement greater than 2-fold. Previous studies have reported that more than 50% of patients experienced tremor power improvement greater than 2-fold [20].

The efficacy and therapy usage can be markedly influenced by the patient selection criteria. For example, a previous study involving a more severe ET population observed a greater improvement in BF-ADL scores with TAPS therapy [20]. Other studies demonstrated that the severity of a patient’s baseline tremor can impact their response to the TAPS therapy, with more severe patients often experiencing greater efficacy [22,34]. Thus, the modest improvement in BF-ADL in the TX arm (1.6) and the TAPS device usage observed in this study could be attributed to the broad inclusion criteria. These criteria did not require a minimum tremor severity or for patients to have used TAPS for more than 90 days or more than 10 stimulation sessions, as required in previous studies [19,20]. Nevertheless, the severe ET subgroup analysis performed in this study suggests that patients experiencing limitations in eating, drinking, and writing based on measures such as BF-ADL may be used to identify patients who most likely respond favorably to therapy. Furthermore, focusing on more severe patients could help allocate healthcare resources to those who are most likely to achieve substantial improvements.

Patients with the most severe tremors had the greatest tremor reduction when they received TAPS therapy, which aligns with previous studies [20,22]. BF-ADL scores also followed a similar pattern displaying a 2.2-point larger improvement in TX compared to SOC in those 65 and older, while observing only a 1.0-point larger improvement in TX compared to SOC for those under 65 (Table 2).

These results are consistent with the growing body of evidence suggesting that TAPS therapy is a safe and effective option for ET patients who are interested in a treatment with less adverse events than current medications and neurosurgical interventions [22,24,35].

### Limitations

There are several limitations associated with this randomized pragmatic clinical trial run during the COVID-19 pandemic that could affect interpretation of these results. First, the open-label design could be confounded with bias, including non-conscious communication of positive biases to patients and biases by study personnel in reporting, data collection, and statistical analysis [36]. Future research is warranted to establish a feasible method to maintain double blinding during remote studies of neuromodulation therapies eliciting sensation [20,37]. Second, patients’ self-reported medication usage data was unavailable. It is estimated that 30 to 50% of patients with ET respond to pharmacotherapy, and use of ET-related medication or polypharmacy may have affected clinical effectiveness and safety of TAPS therapy [7,10]. However, a significant improvement was only observed in patients using TAPS, despite both TX and SOC groups remaining on their medication routine in the study. This finding implies that medication alone may not be sufficient to alleviate tremor in a selected population. Regardless, understanding the interaction between concurrent medications use and effectiveness of TAPS therapy is needed to provide further insights into when to administer therapy to reach optimal therapeutic benefits [8,38,39,40,41]. Third, as in previous studies, patients in the TX arm performed postural holds for measuring tremor only immediately before and after stimulation sessions in this study [20,22,35]. Future studies extending monitoring after stimulation or throughout the day would be beneficial to understanding the duration of therapeutic benefit as well as the variability of hand tremor within a day and between days. Fourth, 49% of patients were missing BF-ADL data (59% for TX, 39% for SOC) at the end of the month, which may produce respondent-selection bias. Similarly, 14% of patients in the SOC and TX arm violated protocol and were therefore included in the mITT but excluded from the PP, which could therefore have respondent-selection bias in the PP results. Fifth, while larger studies with multivariable analysis including comorbidities and other baseline factors would be useful to adjust for potential confounders, the unadjusted data analysis following the prespecified statistical analysis plan was deemed methodologically appropriate. Sixth, because the definition and adoption of SOC in ET is not well established due to a lack of consensus on diagnostic criteria, treatment approaches, complex side effects from pharmacological solutions (30–70% response rate; 20–30% dropout rate from first line medication), and high risk of drug-drug interactions within a highly comorbid and aged population, patients in the TX arm were instructed to continue with their usual care [11,35,40,42,43]. Seventh, patients in the TX and SOC groups were instructed on how to measure their tremor with the wrist-worn device, but adherence to correct device placement and postural hold performance may have confounded the results. Nevertheless, a previous study has shown a significant correlation between clinical tremor ratings and tremor power measured from accelerometry during postural holds [20]. Eighth, using the wrist-worn accelerometry could only provide a proxy assessment of the hand tremor severity, and may not fully capture tremor at the fingers, elbow, or shoulder. Ninth, patients were recruited based on medical claims, and it is well known that medical claims may contain misdiagnoses. This is a frequent challenge for clinical trials using claims databases to identify eligible subjects. This study implemented several measures to mitigate the likelihood of misdiagnosis. For example, the study required 1) at least two medical claims with an ET diagnosis code (ICD-10-CM: G25.0) separately at least seven days apart within the last three years; 2) patients to complete a screening questionnaire confirming their ET symptoms and including a baseline ADL survey to assess the severity of their tremor; and 3) the physician to confirm the patient’s diagnosis of neurology on the prescription form for their TAPS device. While there may have been sampling error due to misdiagnosis, it is important to note that this error would have affected both TX and SOC arms equally because of study randomization. However, this sampling error could limit the generalizability of the study findings to patients without claims that include the ET diagnosis code (ICD-10-CM: G25.0). Tenth, the study only enrolled patients who expressed interest in TAPS therapy and passed the screening; other eligible ET patients may have chosen not to participate. As noted above regarding misdiagnosis, selection bias may also limit the generalization of current findings to a broader ET population. Additionally, the cost of the device makes it prohibitive to enroll patients not interested in TAPS only to have them not use it. Finally, as a pragmatic clinical trial, this study had relatively broad eligibility criteria to allow for generalization to patients in real-world settings. Nevertheless, patients were excluded if they had implanted electronic medical devices (pacemaker, defibrillator, or deep brain stimulator), had undergone ET-related neurosurgeries (thalamotomy, gamma-knife radio surgical thalamotomy, and magnetic resonance-guided focused ultrasound), or intramuscular botulinum toxin. This leaves out some important treatment modalities to which TAPS should be compared in future studies.

## Conclusion

This is the first randomized large-scale pragmatic clinical trial to evaluate unsupervised TAPS use in a real-world home-base setting. The study demonstrated that adding TAPS therapy to existing SOC reduced tremor power and increased improvements in BF-ADL upper limb scores during one month of home use compared to SOC alone. These findings expand and reinforce the prospective and real-world studies suggesting TAPS is a safe and effective treatment option for patients with ET.

## Meeting Presentation

These data have previously been presented as posters at the International Congress of Parkinson’s Disease and Movement Disorders, Madrid, Spain, September 15–18, 2022, and the AMCP 2023, San Antonio, TX, March 21–24, 2023.

## Additional File

The additional file for this article can be found as follows:

10.5334/tohm.798.s1 Supplementary Materials. Table S1–S2 and Figure S1–S3.
