# Supplementary material for: Comparative Effectiveness of Transcutaneous Afferent Patterned Stimulation Therapy for Essential Tremor: A Randomized Pragmatic Clinical Trial
Source: Tremor Other Hyperkinet Mov (N Y). 2023 Oct 16;13:38. doi: 10.5334/tohm.798 (PMC10588491; doi:10.5334/tohm.798)
Supplement: Supplementary Materials. — Table S1–S2 and Figure S1–S3. [file tohm-13-1-798-s1.pdf]

Supplementary Materials

Table S1. Additional sociodemographic and clinical characteristics

Table S2. BF-ADL tasks and overall, at baseline and the end of 1-month

Figure S1. Prospective study design diagram

Figure S2. Tremor and BF-ADL score improvement by tremor severity

Figure S3. Tremor severity distribution at baseline and the end of one month

Analysis of percentages of improved sessions using minimal detectable change

Table S1. Additional sociodemographic and clinical characteristics

**Characteristics**	**Total** (n = 276)	**TX** (n = 133)	**SOC** (n = 143)	***p* value** TX vs SOC
Sociodemographic characteristics				
**Geographic Region**, N (%)				0.47
Midwest	78 (28.26)	33 (24.81)	45 (31.47)	
Northeast	60 (21.74)	33 (24.81)	27 (18.88)	
South	98 (35.51)	46 (34.59)	52 36.36)	
West	19 (14.49)	21 (15.79)	19 (13.29)	
**Urban-rural**, N (%)				0.84
Urban	60 (21.74)	27 (20.30)	33 (23.08)	
Sub-urban	83 (30.07)	40 (30.08)	43 (30.07)	
Rural	133 (48.19)	66 (49.62)	67 (46.85)	
**Household income**, $, Mean (SD)	65641 (21596)	65247 (23542)	66008 (19692)	0.37
**Social vulnerability index (SVI),** Mean (SSD)	0.36 (0.18)	0.35 (0.18)	0.37 (0.18)	0.65
Clinical characteristics				
**Number of comorbidities Group**, N (%)				0.079
≤4	68 (24.64)	38 (28.57)	30 (20.98)	
5-9	148 (53.62)	62 (46.62)	86 (60.14)	
≥10	60 (21.74)	33 (24.81)	27 (18.88)	
**Top 10 most common physical comorbidities**, N (%)				
Pain disorders	229 (82.97)	113 (84.96)	116 (81.12)	0.43
Hyperlipidemia	225 (81.52)	108 (81.20)	117 (81.82)	0.90
Hypertension	190 (68.84)	98 (73.68)	92 (64.34)	0.12

Fatigue and sleep related disorders	124 (44.93)	60 (45.11)	64 (44.76)	0.95
Osteoarthritis	115 (41.67)	51 (38.35)	64 (44.76)	0.28
Obesity	112 (40.58)	61 (45.86)	51 (35.66)	0.088
Chronic pulmonary disease	77 (27.90)	33 (24.81)	44 (30.77)	0.27
Diabetes mellitus	70 (25.36)	32 (24.06)	38 (26.57)	0.63
Ischemic heart disease	65 (23.55)	33 (24.81)	32 (22.38)	0.63
Chronic kidney disease	49 (17.75)	20 (15.04)	29 (20.28)	0.25
Fall	49 (17.75)	29 (21.80)	20 (13.99)	0.11
Cerebrovascular disease	42 (15.22)	17 (12.78)	25 (17.48)	0.28
Cancer (malignant)	39 (14.13)	19 (14.29)	20 (13.99)	0.94
**Medication use**, N (%)				
Psychiatric medications				
Antidepressants	77 (27.90)	34 (25.56)	43 (30.07)	0.40
Anxiolytics	38 (13.77)	19 (14.29)	19 (13.29)	0.81
Antipsychotics/Antimanic agents	12 (4.35)	7 (5.26)	5 (3.50)	0.56
Hypnotics	10 (3.62)	6 (4.51)	4 (2.80)	0.53

Improvement in Individual BF-ADL Tasks

Table S2 presents the improvements in individual BF-ADL tasks, as a supplement to the results presented for the BF-ADL score in the main manuscript.

Table S2. BF-ADL tasks and overall, at baseline and the end of 1-month

**BF-ADL task**[1]	**TX (n = 133)**			**SOC (n = 143)**		
	Baseline	1-month	*P* value[3]	Baseline	1-month	*P* value[3]
	Mean (SD)	Mean (SD)		Mean (SD)	Mean (SD)	
Use a spoon to drink soup	2.41 (0.79)	2.09 (0.89)	0.0031	2.35 (0.80)	2.32 (0.87)	0.8836
Hold a cup of tea	2.28 (0.86)	2.00 (0.77)	0.0075	2.33 (0.77)	2.30 (0.84)	0.7616
Pour milk from a bottle	2.06 (0.85)	1.75 (0.77)	0.0036	2.00 (0.76)	2.02 (0.80)	0.8439
Dial a telephone	1.90 (0.83)	1.57 (0.72)	0.0018	1.79 (0.84)	1.75 (0.85)	0.6439
Pick up change	2.03 (0.87)	1.84 (0.87)	0.0954	1.99 (0.80)	2.03 (0.87)	0.8505
Insert an electric plug	1.81 (0.79)	1.57 (0.72)	0.0182	1.90 (0.86)	1.88 (0.83)	0.9601
Unlock front door	1.90 (0.74)	1.67 (0.76)	0.0156	1.92 (0.75)	1.87 (0.81)	0.4562
Write a letter	2.89 (0.83)	2.71 (0.80)	0.1021	2.91 (0.85)	2.90 (0.92)	0.9566
Total score	17.23 (5.14)	14.74 (4.71)	0.0006	17.07 (4.86)	17.12 (5.36)	0.5548
Overall average[2]	2.16 (0.63)	1.90 (0.60)	0.0008	2.15 (0.61)	2.13 (0.64)	0.6976

1. Each BF-ADL task rated 1–4 by patient (1 = able to do the activity without difficulty, 2 = able to do the activity with little effort, 3 = able to do the activity with a lot of effort, and 4 = cannot do the activity by yourself)
2. Mean all BF-ADL score (8 tasks).
3. Holm-Bonferroni corrections for multiple hypothesis testing

12-Month Study Design

This manuscript describes the randomized portion of a study, which took place during the first month of a 12-month study (Figure S1). After completing a month in the SOC arm, all SOC arm patients crossed into the TX arm for 11 months of TAPS therapy. All patients had completed the first month of the study as of March 2023 and all patients are anticipated to have completed the study in March 2024.

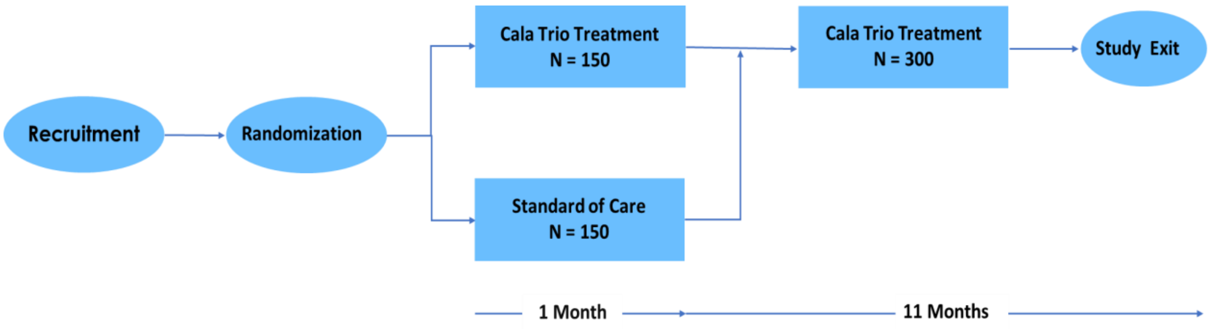

Figure S1. Prospective study design diagram.

Data Splits by Tremor Power Quartiles

To assess the relationship between tremor severity and the degree of tremor improvement, tremor improvement in the TX arm was assessed by binning the patient's tremor power before stimulation into quartiles. Patients were divided into quartiles, representing low (0-25%), medium (25-75%), and high tremor severity (75-100%), and the tremor power improvement ratio (TPIR) and change in BF-ADL from baseline to one month were evaluated for each quartile (Figure S2).

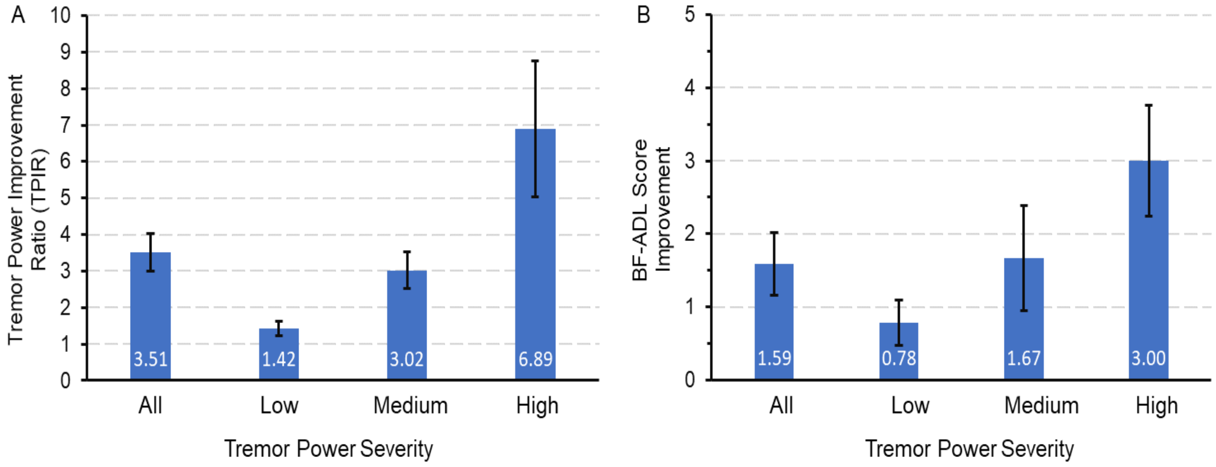

Figure S2. Tremor and BF-ADL score improvement by tremor severity. (A) Tremor power improvement (TPIR) stratified by tremor severity ($p < 0.0001$). **(B)** BF-ADL improvement stratified by tremor severity ($p = 0.2081$). Tremor severity categories were defined as pre-stimulation tremor power quartiles (low severity (0-25%), medium severity (25-75%) and high severity (75-100%)). Data is presented as geometric mean ± standard error.

Data Splits by BF-ADL score

To assess the response in patients with more severe tremor, patients were classified by severity of their BF-ADL scores at baseline and one month as follows: “None” (BF-ADL score: 8), “Mild” (9–16), “Moderate” (17–24), or “Severe” (25–32). The responder rate for patients with a BF-ADL score of “Moderate” or “Severe” at baseline whose BF-ADL score improved to a rating of “Mild” or “None” at the end of the month was assessed**.** Sixty-one percent of patients whose baseline BF-ADL score was rated “Moderate” or “Severe” improved to “Mild” or “No tremor” in the TX arm at one month, while only 17% patients observed the same improvement in the SOC arm (p < 0.0001) (Figure S3).

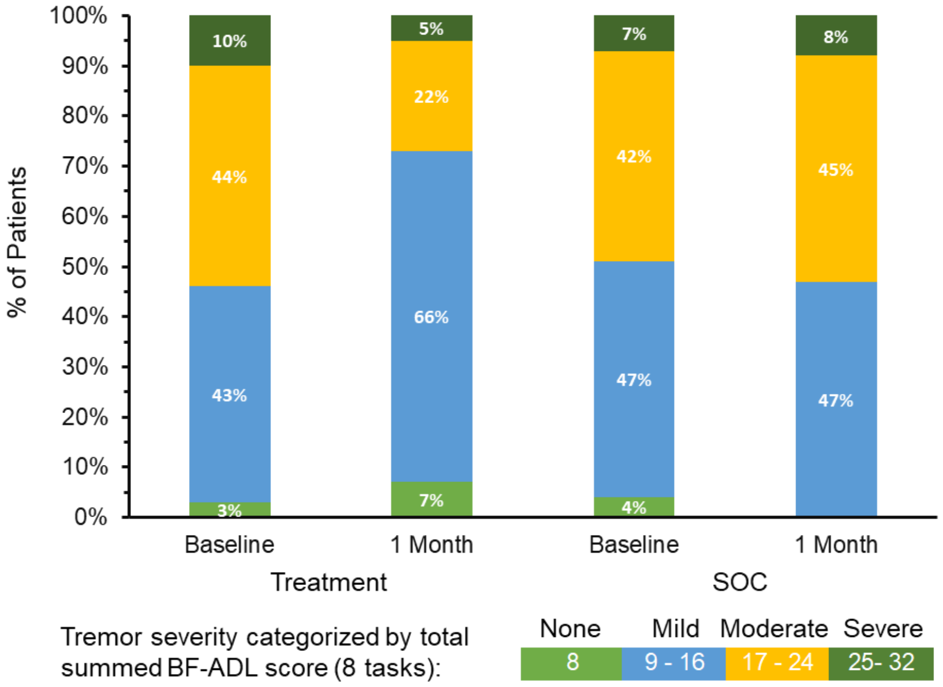

Figure S3. Tremor severity distribution at baseline and the end of one month. The severity was defined as “None” (BF-ADL 8 task score: 0-8), “Mild” (9–16), “Moderate” (17–24), or “Severe” (25–32) based on the BF-ADL upper limb scores. In the TX arm, 61% of patients with tremor ratings of Moderate or Severe at baseline were classified as Mild or None by the end of one month. Only 17% of patients experienced the same improvement in the SOC arm (p < 0.0001).

Analysis of percentages of improved sessions using minimal detectable change

An additional analysis was performed to estimate the minimal detectable change (MDC) in the TX arm, based on pre-stimulation tremor power. This helps to account for possible measurement noise when reporting the percentage of sessions with tremor power improvement ratio greater than 1. Prior research indicates that the MDC can be derived from the within-subjects residual mean squared error (representing within-subjects variability) in a repeated-measures analysis of variance (ANOVA) (1,2). However, given the pragmatic nature of this study, using a repeated-measures ANOVA seems unsuitable due to its unbalanced data structure, such as tremor power measurement varied across patients. On the other hand, mixed models can cater to such unbalanced design and incorporate all existing data points (3).

A mixed effects model was constructed using the log10-transformed post-stimulation tremor power as the dependent variable. The log10-transformed pre-stimulation tremor power served as the fixed effect, while each patient was considered a random effect. From this model, the 95% confidence interval (CI) for the log10-transformed pre-stimulation tremor power was determined using a parametric bootstrap approach with 1,000 repetitions. The MDC was defined as the absolute difference between the 95% CI. An improvement in each session was identified when the change in pre-and post-stimulation tremor power (both log10-transformed) exceeded the MDC.

The effect of log10-transformed pre-stimulation tremor power was statistically significant and positive (beta = 0.30; 95% CI, [0.27, 0.33]; $t(4480) = 20.23$; $p < 0.001$). The model's intercept (log10-transformed pre-stimulation tremor power equal to 0) was at –1.30 (95% CI, [-1.39, -1.21]; $t(4480) = -28.51$; $p < 0.001$). The MDC value was 0.06, equivalent to the original tremor power improvement ratio at 1.15 (back transformed from log10-transformation value). The results indicate that 75.4% of patients experienced at least 50% of sessions that were improved above MDC, and the median percentage of improved sessions was 64.2% (IQR, 24.0%; mean, 64.2%; SD, 18.8%) across the TX arm.

References

1. Weir JP. Quantifying Test-Retest Reliability Using the Intraclass Correlation Coefficient and the SEM. Journal of Strength and Conditioning Research. 2005;19(1):231–240.
2. Elble RJ, McNames J. Using Portable Transducers to Measure Tremor Severity. Tremor and Other Hyperkinetic Movements. 2016;6(0):375.
3. Detry MA, Ma Y. Analyzing Repeated Measurements Using Mixed Models. JAMA. 2016;315(4):407-408.
